# APATHY IN NURSING HOME RESIDENTS WITH DEMENTIA: ANALYSIS OF WITHIN- AND BETWEEN-PERSON DIFFERENCES

**DOI:** 10.1093/geroni/igad104.2284

**Published:** 2023-12-21

**Authors:** Ying-Ling Jao, Yo-Jen Liao, Diane Berish, Jacqueline Mogle, Helen Kales, Marie Boltz

**Affiliations:** Pennsylvania State University, State College, Pennsylvania, United States; Penn State University, State College, Pennsylvania, United States; The Pennsylvania State University, University Park, Pennsylvania, United States; Clemson University, Clemson, South Carolina, United States; University of California, Davis Health, Davis, California, United States; Pennsylvania State University, State College, Pennsylvania, United States

## Abstract

Apathy commonly occurs in persons with dementia, yet it is often overlooked in nursing homes. It is unclear how apathy symptoms differ within- and between-individuals. This observational study examined the differences in apathy among nursing home residents with dementia. Three videos, 3-10 minutes each, were recorded for each resident to capture caregiving interactions between caregivers and residents with dementia and apathy. Apathy levels were rated second-by-second by trained researchers via video observations using the Person-Environment Apathy Rating-Apathy subscale (PEAR-Apathy). The total PEAR-Apathy score ranges from 6-24, with higher scores indicating higher apathy levels. A total of 17 residents were enrolled from three nursing homes in Pennsylvania. Correlations and multilevel modeling were used for analyses. PEAR-Apathy score was 16.2 on average. Within-video average variability in apathy was 2.28 (range=0.47-4.24). Across-video within-person results showed average variability was 2.27, and the range between people was 1.36 to 3.31. Residents’ baseline apathy and depression, caregiver age, dementia care experience, and experience with the resident were not significantly correlated with apathy variability. Results from multilevel modeling showed that 11.2% of the within-person variance in apathy was accounted for by resident differences. After accounting for the clustering at the resident level, age was significantly associated with apathy variability (t=3.31, p=0.002). Results suggest that residents with dementia have moderate differences in apathy variability. Additionally, variability in apathy tended to increase with age. Future research may further examine factors contributing to individual differences in apathy and develop interventions to minimize apathy in nursing home residents with dementia.

